# Genome-wide differentially methylated genes associated with post-traumatic stress disorder in female rape survivors

**DOI:** 10.4102/sajpsychiatry.v24i0.1311

**Published:** 2018-09-24

**Authors:** Jani Nöthling, Soraya Seedat, Naeemah Abrahams, Sian Hemmings

**Affiliations:** 1 Department of Psychiatry, Stellenbosch University, South Africa; 2 South African Medical Research Council, South Africa; 3 Division of Molecular Biology and Human Genetics, Stellenbosch University, South Africa

## Abstract

**Introduction:**

Alterations to the epigenome in response to psychological trauma have been reported as a mechanism mediating gene and environmental interaction. Differentially methylated genes involved in the biological pathways associated with the adverse phenotypic behavioural presentations in post-traumatic stress disorder (PTSD) have previously been identified. However, the majority of studies have focussed on differential methylation of single candidate genes in participants exposed to heterogeneous index traumas. The objective of this study was to identify genome-wide differences in methylation profiles of a group of women exposed to rape, with and without PTSD.

**Methods:**

Female isiZulu participants (*n* = 48) between 18 and 40 years of age, who reported an incident of rape within the previous 20 days, were recruited from three Thuthuzela care centres and a crisis clinic in Durban, KwaZulu-Natal. Rape-exposed participants with and without PTSD were matched on HIV status, age, childhood maltreatment and other lifetime trauma exposure, body mass index and smoking status. DNA was extracted from peripheral blood and analysed using the Illumina Epic BeadChip microarray. Logistic regression models, adjusting for multiple comparisons, were used to identify differentially methylated genes in participants with and without PTSD at 3 months post-rape.

**Results:**

Four hundred twenty-three differentially methylated genomic regions were associated with PTSD status. Paired Box 8 (PAX8), encompassing eight CpG sites (*p* = 9.14E-20 and Zinc Finger Protein 57 [ZFP57]) and 19 CpG sites (*p* = 4.84E-18) were among the top 20 genomic regions significantly associated with PTSD in this data set, which were previously found to be associated with PTSD in other traumatised cohorts.

**Conclusion:**

PAX8 may be involved in PTSD symptoms related to sleeping difficulties and ZFP57 is believed to be involved in susceptibility to stress governed by differential methylation in hippocampal cells. This is the first study, to our knowledge, to investigate genome-wide profiles of women exposed to rape in Africa. Confirmation of these findings will require replication in larger cohorts.

